# Chemical Analysis of *Eruca sativa* Ethanolic Extract and Its Effects on Hyperuricaemia

**DOI:** 10.3390/molecules27051506

**Published:** 2022-02-23

**Authors:** Arthur Ferrari Teixeira, Jacqueline de Souza, Douglas Daniel Dophine, José Dias de Souza Filho, Dênia Antunes Saúde-Guimarães

**Affiliations:** 1 Laboratório de Plantas Medicinais (LAPLAMED), Programa de Pós-Graduação em Ciências Farmacêuticas (CiPharma), Universidade Federal de Ouro Preto, Ouro Preto 354000-000, Brazil; arthurfarmaufop@gmail.com (A.F.T.); douglasdophine@gmail.com (D.D.D.); 2 Laboratório de Controle de Qualidade (LCQ), Universidade Federal de Ouro Preto, Ouro Preto 354000-000, Brazil; jacsouza@ufop.edu.br; 3 Laboratório Multiusuário de Caracterização de Moléculas (LMCM), Programa de Pós-Graduação em Ciências Farmacêuticas (CiPharma), Universidade Federal de Ouro Preto, Ouro Preto 354000-000, Brazil; jose.dsf@ufop.edu.br

**Keywords:** glucosinolate, glucosylated flavonols, rocket, validation, NMR, UHPLC/ESI/QTOF, hyperuricaemia

## Abstract

In vivo assays and chemical analyses were performed on the ethanolic extract from leaves of *Eruca sativa*. UHPLC-ESI-QTOF analysis confirmed the presence of glucosinolates and flavonol glucosides. The major flavonoid of the ethanolic extract, kaempferol-3,4′-di-*O*-β-glucoside, was isolated, a HPLC-DAD method developed and validated to quantify its content in the extract. In vivo experiments were carried out on Wistar rats with hyperuricaemia induced by potassium oxonate and uric acid. A hypouricaemic effect was observed in hyperuricaemic Wistar rats treated with ethanolic extract at dose of 125 mg/kg and kaempferol-3,4′-di-*O*-β-glucoside at dose of 10 mg/kg. The main anti-hyperuricaemic mechanism observed in the extract was uricosuric. Kaempferol-3,4′-di-*O*-β-glucoside was identified as an important component responsible for the total activity of the ethanolic extract and was considered as a good chemical and biological marker of the ethanolic extract of *E. sativa.* The obtained results indicated the potential of *E. sativa* in the treatment of hyperuricaemia and its comorbidities.

## 1. Introduction

Hyperuricaemia occurs due to the overproduction of uric acid or its insufficient excretion. The high levels of uric acid in the blood cause various damages to patients, such as the development of gout, endothelial dysfunction, hypertension, diabetes, and heart and kidney diseases [1].

Natural products are considered a great source of bioactive substances, as they have a rich chemical profile. The potential of several plant species in relation to anti-hyperuricaemic therapy has already been demonstrated [2].

Rocket, *Eruca sativa* Miller, is a vegetable widespread in several regions of the world and commonly used in food. It is consumed pure in salads, cooked with meat, processed in pesto or in the form of extracts. Rocket in Brazil is usually grown in open fields or in a hydroponic system [3]. Animal models have already shown that rocket is associated with several biological activities, such as antihypertensive, nephroprotective and antidiabetic activities [4,5,6]. Such biological activities are desirable since hyperuricaemia is closely related to endothelial dysfunction and is pointed out as a causal factor of diseases such as hypertension and diabetes [1].

Looking from the opposite point of view, the fact that *E. sativa* species demonstrate benefits in hypertension and diabetes may be an important lead that this plant also acts as an anti-hyperuricaemic agent. The chemical constitution of rocket has been well studied and many biological activities have been attributed to isolated compounds, such as glucosinolates and flavonoids [6,7]. However, little is known about the chemistry of *Eruca sativa* grown in Brazil. Studies on its therapeutic effects are limited, despite the fact that this plant has several ethnopharmacological uses reported in other countries.

The discovery of anti-hyperuricaemic effects in *E. sativa* would draw attention to its potential in the treatment of hyperuricaemia and its associated comorbidities.

## 2. Results and Discussion

### 2.1. Qualitative HPLC Analyses of EE from E. sativa Leaves

#### 2.1.1. Qualitative HPLC-DAD Analysis

**Figure 1:**
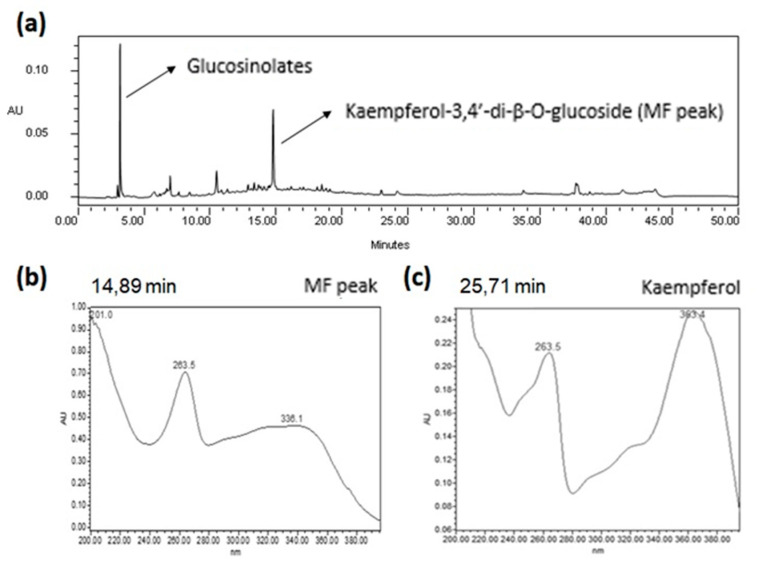
Chromatographic profile of ethanolic extract of *Eruca sativa* leaves and comparison of UV-spectra of MF peak and kaempferol. (**a**) Chromatogram of the ethanolic extract of *Eruca sativa* leaves obtained by HPLC-DAD at 264 nm. (**b**) UV spectra of MF peak extracted from chromatogram of Figure 1a. (**c**) UV spectra of kaempferol standard injected independently at the same chromatographic conditions of MF peak.

Fingerprinting of the ethanolic extract from leaves of *Eruca sativa* (EE) obtained by HPLC-DAD is shown in Figure 1. Peaks along the chromatogram are concentrated between Rt = 4.50 and 15.00 min, indicating a polar bias of substances in the extract. Several minor peaks were seen in this region and two major peaks (4.81 and 14.89 min) stood out. Analysis of the major peaks in the UV spectra showed behaviour characteristics of a glucosinolate and a flavonol, respectively [8,9]. In order to acquire more structural data of the major flavonoid (MF), the UV spectra of kaempferol standard was compared under the same conditions (Figure 1). No differences were observed in relation to band II, indicating that both structures contain a kaempferol aglycone moiety. In relation to band I, a hypsochromic effect of 27 nm from kaempferol to MF was noticed, suggesting substitutions of hydroxyl groups at positions 3 and 4′ [8]. This suggestion was further confirmed by HPLC-DAD analysis, by showing that flavonoid isolated from EE (YP) matched in retention time and UV spectra with the MF in EE. The relative area of the MF in YP corresponded to 96% of the chromatogram, suggesting a high degree of purity.

#### 2.1.2. Qualitative UHPLC-ESI-QTOF Analysis

Mass spectral (MS) data obtained by UHPLC-ESI-QTOF was used to gather further knowledge about EE composition, and it is summarised in Table 1. It was possible to identify nine substances: kaempferol-3-*O-*β-glucoside, kaempferol-3,4′-di-*O*-β-glucoside, kaempferol-3-*O-*(2-sinapoyl- β-glucoside)-4′-*O*-glucoside, glucosativin glucosinolate, glucoraphanin glucosinolate, leucine, tryptophan, angustione and erucamide by comparing their molecular ions and mass fragments with the literature and mass banks available.

In the spectrum of flavonoids-*O*-glucosylated, the molecular ion [M+H]^+^ is normally of low intensity, and the loss of a sugar molecule is evident. The product is a prominent Y0+ aglycon ion. In the case of flavonoid-di-*O*-glucosylates, an intermediate Y1+ corresponding to the loss of a sugar molecule is observed [15]. MS analyses showed the presence of a molecular ion [M+H]^+^ of 611 m/z at 12.6 min and the formation of fragments consistent with losses of two hexose fragments, confirming the identification of a diglucoside of kaempferol. At 15.1 min, a molecular ion [M+H]^+^ of 449 m/z and its secondary fragmentation, compatible with one loss of a hexose, allowed the identification of a monoglucoside of kaempferol. Comparison with the literature strongly suggested the identity of these flavonoids to be kaempferol-3,4′-di-*O*-β-glucoside and kaempferol-3-*O-*β-glucoside [13].

Cuyckens et al. [14] studied the fragmentation of acylated flavonol-*O*-glucosides and identified fragments of groups such as feruloyl, sinapoyl, coumaroyl and benzoyl, among others. It was found that these structures can be observed in the mass spectra in the positive mode at low-energy CID conditions. This knowledge enabled the identification of an acylated flavonol-*O*-glucoside in the EE. The molecular ion peak with m/z 817 was observed at Rt = 16.2 min. Secondary fragmentations showed the loss of a glucose fragment (m/z 162) and the formation of a fragment with m/z 655. In sequence, a loss of a fragment of m/z 286, characteristic of a kaempferol aglycone, and the formation of a fragment with m/z 369 associated with a sinapoyl-hexose group, were observed. Another loss of a hexose (m/z 162) led to the formation of a fragment with m/z 207 of the sinapoyl group. These results suggested the peak identity with Rt = 16.2 min as kaempferol-3-(2-sinapoyl-β-glucoside)-4′-*O*-glucoside.

Glucosinolate identification was sustained by the observation of fragments with m/z 328.1384 and 166.0861 characteristic of glucosativin glucosinolate (Rt = 1.0 min), and fragments of m/z 438.055, 358.0987 and 196.0457 characteristic of glucoraphanine (Rt = 1.8 min) [10,11,12].

Aminoacids such leucine (182 m/z, 268 m/z and 132 m/z) and tryptophan (188 m/z, 146 m/z and 205 m/z) were identified by comparison with the mass bank of Fiocruz Institute at 1.4 and 4.2 min, respectively. Previous work by Bell et al. [16] quantified amino acids in *E. sativa* varieties, finding leucine levels in the range of 2.6–3.5 µg/g. Furthermore, it is known that these amino acids (leucine and tryptophan) are biosynthetic precursors of glucosinolates [17]. Other substances, such as angustione (197 m/z and 179 m/z) and erucamide (338 m/z), an amide derived from erucic acid, were also identified by comparison with the mass bank of the Fiocruz Institute at 14.5 and 46.4 min. It was reported that erucic acid is normally found in high levels in *E. sativa* seeds [18].

### 2.2. Full NMR Structural Characterisation of Kaempferol-3,4′-di-O-β-Glucoside 

**Figure 2:**
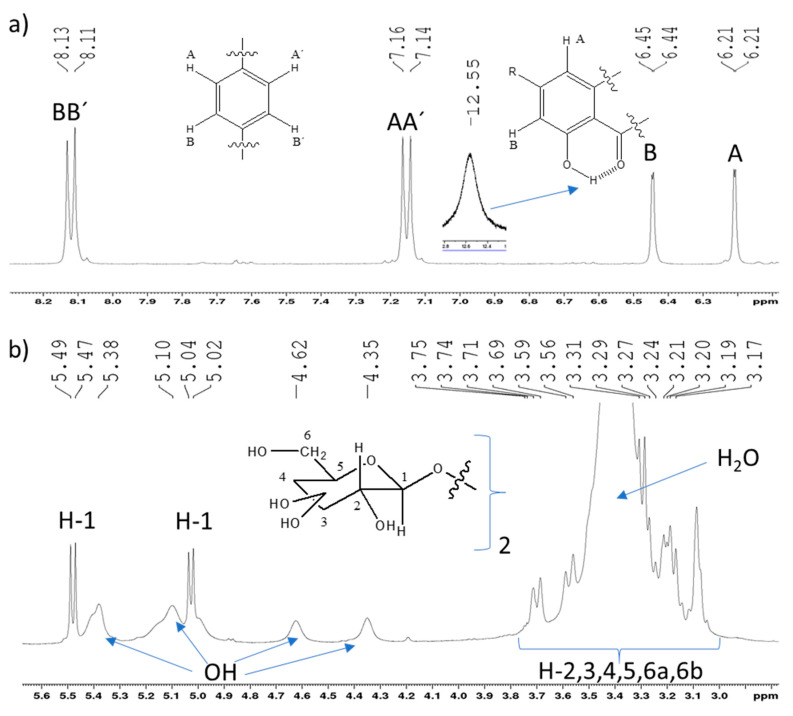

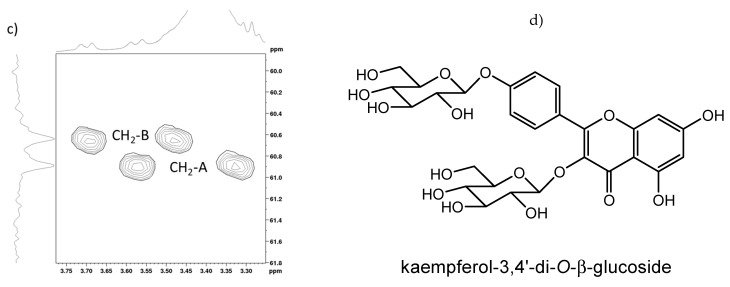
Diglucoside nature of the flavonoid. (**a**) Aromatic region; (**b**) sugar moieties region; (**c**) HSQC expansion of the pyranosidic protons focusing correlations of the two methylenic groups (DEPT135 in vertical F1 projection) and (**d**) complete chemical structure.

Notably, the ^1^H NMR spectrum profile revealed the flavonoidic nature of this compound because of the apparent AA’BB’ doublets and meta-coupled AB system (J 1.76 Hz) signals in the aromatic region (Figure 2a). At δ 5.03 and δ 5.48 are seen two well resolved doublets (J 7.25 Hz and 7.50 Hz, respectively) that can be assigned to two anomeric protons (Figure 2b). Despite the lack of resolution in the 3.0–3.8 ppm region (Figure 2b) due to the water signal from the solvent, one can confirm the presence of the two pyranosidic units from the HSQC contour map by the two double correlations due to two methylenic groups, shown in Figure 2c. In the 4.2–5.6 ppm region, one can observe broad signals of hydroxyl groups due to the effect of hydrogen bonds with the solvent (Figure 2b). Moreover, a very unshielded proton signal is registered at δ 12.55, indicating the presence of a quelated hydroxy group in the molecule (Figure 2a).

As one can see, all similar pyranosidic carbon signals are paired. Figure 2c,d shows the carbon and DEPT135 spectra as the F1 projection. The ^13^C NMR spectrum exhibits 25 signals (Table 2) that are compatible with the 27 predicted carbons in the fragments shown in Figure 2d, which indicates a structural formula containing an aglycone-type kaempferol and two sugar units.

**Figure 3:**
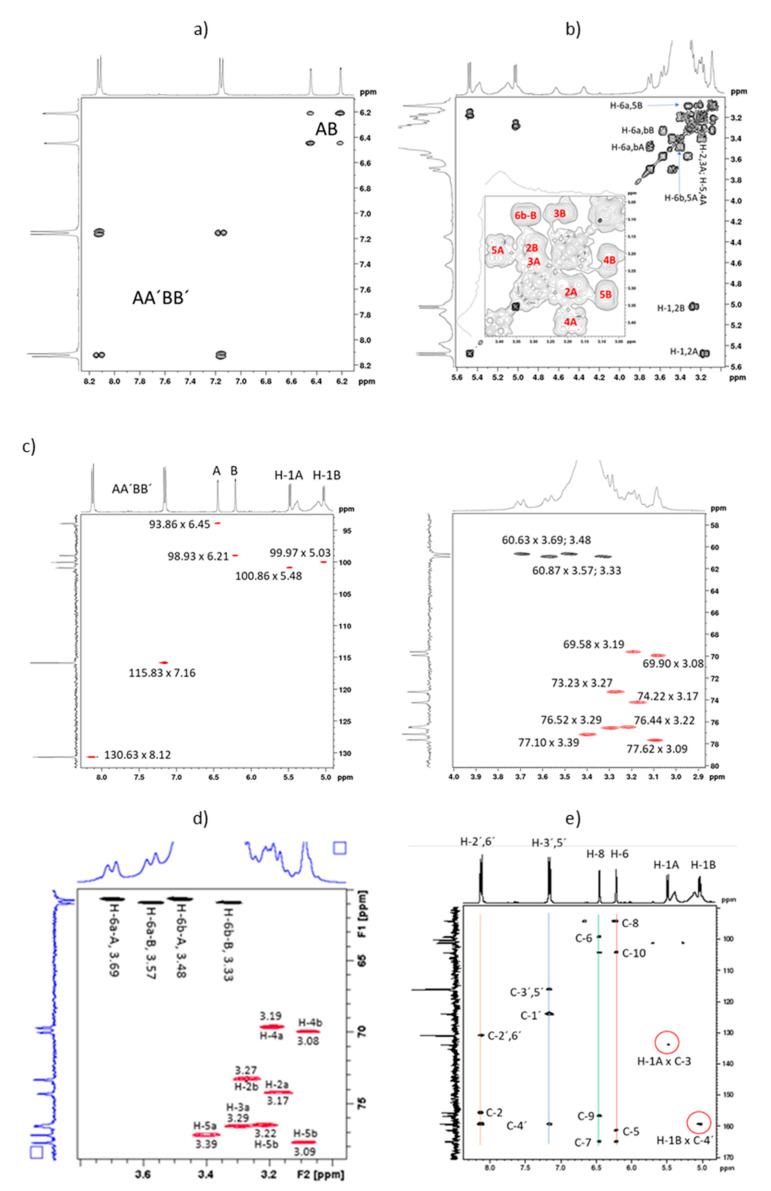
(**a**) COSY expanded contour plot of the aromatic region showing the AA’BB’ and AB coupling systems; (**b**) COSY expanded contour plot of the pyranosidic region; (**c**) HSQC expanded contour plot of the pyranosidic region showing the precise chemical shifts; (**d**) assignments of the pyranosidic unities and (**e**) HMBC expanded contour plot showing the kaempferol 3 and 4′ glucosilated positions.

The homonuclear 2 D COSY experiment produced a contour plot (Figure 3a) where the AA’BB’ and AB aromatic correlations could be promptly assigned. The 3.0–3.8 ppm region shows an extreme overlapping of the pyranosidic proton signals, and one can observe two correlations for each anomeric hydrogen (Figure 3b). This is probably due to the presence of rotamers. The chemical shifts of H-2 A (δ 3.17) and H-2B (δ 3.27) as well as those of H-5 A (δ 3.39) and H-5B (δ 3.09) are assigned to H-1 A,B and H-6 A,B, respectively. H-5B and H-4B are very close in chemical shift (δ 3.09 and 3.08, respectively) with correlations over the diagonal in the 2 D COSY contour plot. H-4B exhibits its coupling to H-3B (δ 3.22) by a discrete correlation (Figure 3b). The assignment of the correlations in this region was supported by the edited HSQC contour plots shown in Figure 3c,d.

The anomeric proton signals as doublets at δ 5.48 (H-1 A, J 7.50 Hz) and δ 5.03 (H-1B, J 7.25 Hz) are due to β-glucosides because of the magnitude of the vicinal coupling constants. The very elegant HMBC experiment determined the location of each sugar moiety A and B by the long-range coupling strategy to establish the spin systems. Figure 3e confirms the position of sugar moieties at position 3 and 4′ by the presence of the correlations between the chemical shifts δ 5.48 × 133.78 (H-1 A × C4′) and δ 5.03 × 159.24 (H-1B × C3). The non-hydrogenated carbons are readily assigned via three- and two-bond couplings.

To the best of our knowledge, this is the first complete assignment of the NMR data of kaempferol-3,4′-di-*O*-β-glucoside (Table 2). The proton signal multiplicities of the pyranosidic region could not be extracted because of the high degree of overlapping and the existence of rotamers.

### 2.3. Quantification of the Major Flavonoid Kaempferol-3,4′-di-O-β-glucoside in the EE of E. sativa Leaves by the Validated HPLC-DAD Method

Concentration of kaempferol-3,4′-di-*O*-β-glucoside quantified in sample solutions (*n* = 3) was 0.00716 ± 0.00021 mg/mL, which was above the LQ (0.0059 mg/mL). Therefore, it was calculated that the EE tested in rats contained 3.5 ± 0.283 g rutin equivalents/kg EE. In comparison, Martínez-Sánchez et al. [19] reported a content of kaempferol-3,4′-di-*O*-β-glucoside of 0.978 g/kg of fresh plant, while other flavonoids presented contents 10-fold lower. Other work carried out with several varieties of *E. sativa* showed, by means of statistical analysis (principal component analysis—PCA), that there is a strong tendency in varieties of *E. sativa* to accumulate flavonols with a kaempferol scaffold, especially kaempferol-3,4′-di-*O*-β-glucoside, with some cultivars presenting contents of 1.1 g/kg of kaempferol-3,4′-di-*O*-β-glucoside. Kaempferol-3-*O*-glucoside is found in lower amounts than kaempferol-3,4′-di-*O*-β-glucoside in most varieties, demonstrating that the biosynthesis of the diglucosylated kaempferol is favoured in this species [20]. That fact justifies choosing kaempferol-3,4′-di-*O*-β-glucoside as a chemical marker in the EE of *E. sativa* leaves.

### 2.4. Effects of Ethanolic Extract of E. sativa Leaves on Hyperuricaemia-Induced Rats

**Figure 4:**
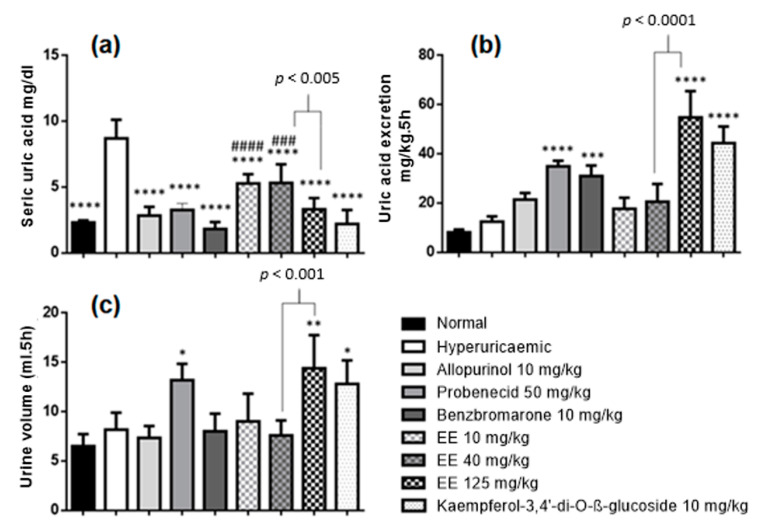
Biological effects in hyperuricaemic Wistar rats after treatments with E. sativa ethanolic extracts and positive controls allopurinol, probenecid and benzobromarone (**a**) Seric concentrations of uric acid (**b**) Excretion of uric acid (**c**) Urine volume excreted after 5 h of the treatment. *** *p* < 0.001 vs. hyperuricaemic control group; ### *p* < 0.001 vs. normal control group; #### *p* < 0.0001 vs. normal control group; **** *p* < 0.0001 vs. hyperuricaemic control group; *** *p* < 0.001 vs. hyperuricaemic control group; ** *p* < 0.01 vs. hyperuricaemic control group; * *p* < 0.05 vs. hyperuricaemic control group (*n* = 6, One-way ANOVA followed by the Dunnet test).

The serum uric acid level in the hyperuricaemic control group (8 ± 1.415 mg/dL) was significantly higher than in the normal group (2 ± 0.1630 mg/dL), attesting that the model was effective in increasing uric acid levels in the blood (Figure 4a). The hypouricaemic effect was associated with groups that had serum uric acid levels lower than in the hyperuricaemic group. Positive controls (allopurinol, probenecid and benzbromarone) were effective in reducing serum hyperuricaemia to normal levels, as the concentrations with these treatments showed significant differences with the hyperuricaemic control group and no differences from the normal control group. Animals treated with EE at a dose of 125 mg/kg showed serum uric acid levels similar to the normal control. Treatments with EE at doses of 40 and 10 mg/kg were statistically equivalent and demonstrated a hypouricaemic effect; however, this effect was not able to reduce uricaemia to normal levels. The flavonoid isolated from the EE (kaempferol-3,4′-di-*O*-β-glucoside) at a dose of 10 mg/kg was effective in reducing the uricaemia of hyperuricaemic rats to normal levels. The anti-hyperuricaemic activity of EE (125 mg/kg) and kaempferol-3,4′-di-*O*-β-glucoside (10 mg/kg) were statistically equal to positive controls (allopurinol, probenecid and benzbromarone).

Allopurinol is an inhibitor of xanthine oxidase (uricostatic activity), and acts by decreasing the production of uric acid in the liver [21]. The residual activity of xanthine oxidase was evaluated, and treatment with allopurinol promoted an inhibition of 54.72% in relation to the hyperuricaemic group. None of the other treatments managed to promote xanthine oxidase inhibition, suggesting that EE and kaempferol-3,4′-di-*O*-β-glucoside do not promote uricostatic activity.

The uricosuric effect of the treatments were evaluated, and the results are illustrated in Figure 4b. Treatments with probenecid (34 ± 2.304 mg/kg 5 h) and benzbromarone (30 ± 4.253 mg/kg 5 h) were effective in exerting a uricosuric effect in the rats. Treatments with EE and with kaempferol-3,4′-di-*O*-β-glucoside exerted uricosuric effects. The amount of uric acid excreted by the rats when receiving EE at a dose of 125 mg/kg was 54 ± 10.66 mg/kg 5 h (Figure 4b), which was statistically more effective than the values obtained in the treatments with probenecid and benzbromarone (Tukey test, *p* ≤ 0.05). Treatment with kaempferol-3,4′-di-*O*-β-glucoside showed 44.00 ± 6.842 mg/kg 5 h a better uricosuric effect than benzbromarone (Tukey test *p* ≤ 0.05), but equivalent to probenecid. Other treatments (EE at 40 and 10 mg/kg) did not exert an uricosuric effect. These data suggest that at doses of 125 mg/kg of EE and 10 mg/kg of kaempferol-3,4′-di-*O*-β-glucoside, the uricosuric effect is the main mechanism responsible for the hypouricaemic effect. However, it was impossible to explain why a hypouricaemic effect was seen on treatment with EE at 10 and 40 mg/kg, given that these doses did not promote uricosuric and uricostatic effects. This suggests that other mechanisms are contributing to the anti-hyperuricaemic activity of these treatments. Furthermore, it was seen that kaempferol-3,4′-di-*O*-β-glucoside, the major component of EE, plays a crucial role in the overall anti-hyperuricaemic activity of the EE and can be seen as a biological marker for uricosuric activity.

Additionally, the treatments that exerted an uricosuric effect (EE 125 mg/kg; kaempferol-3,4′-di-*O*-β-glucoside), also showed a diuretic effect in relation to the hyperuricaemic and normal groups (Figure 4c). This is particularly convenient because one of the problems that can appear with the use of uricosuric substances is urolithiasis, i.e., when supersaturation of uric acid takes place leading to precipitation of urate crystals in the kidneys, urethra or bladder [22]. Therefore, the diuretic effects observed in treatments with EE of *E. sativa* and kaempferol-3,4′-di-*O*-β-glucoside have a positive and synergic effect because they aid in urinary excretion and minimise possible episodes of urolithiasis.

It was observed that the water balance was negative in hyperuricaemic animals. The fact that these animals were dehydrated could have underestimated the results obtained by treatments that showed a uricosuric response in this model. Such a proposition may indicate that the uricosuric and diuretic responses may have been greater than actually shown.

Flavonoids have been associated with anti-hyperuricaemic effects. It has been reported that myricetin, quercetin and kaempferol aglycones inhibit xanthine oxidase in vitro, whereas rutin, the glycosylated form of quercetin, does not [23]. A study by Haidari et al. [24] showed that hyperuricaemic Wistar rats treated with kaempferol (5 mg/kg) had a significant reduction in serum uric acid levels and hepatic xanthine oxidase and xanthine dehydrogenase activities were inhibited. Similar results were found by Zhu et al. [25], who explored the anti-hyperuricaemic effects in mice of flavonoids isolated from *Biota orientalis* by different routes of administration. When administered orally, quercetin, rutin and *B. orientalis* extract demonstrated significant inhibition of xanthine oxidase and xanthine dehydrogenase. In contrast, when these same treatments were administered intraperitoneally, quercetin and the extract demonstrated a subtle reduction in hyperuricaemia, while rutin had no hypouricaemic effect. It is known that the pharmacokinetics of flavonoids varies by adding sugars to the structure [26]. In the present study, kaempferol-3,4′-di-*O*-β-glucoside administered intraperitoneally to rats demonstrated a potent hypouricaemic effect at a dose of 10 mg/kg. This suggests that hexoses in positions 3 and 4′ give a different pharmacological profile to kaempferol-3,4′-di-*O*-β-glucoside, since the treatment was remarkably effective intraperitoneally and one of the mechanisms involved was uricosuric. Previous studies [27] have suggested that the presence of hydroxyl groups at positions C3, C5, C7 and C-4′ of the chemical structure of flavonoids are important for XO-inhibiting and antihyperuricaemic activity. This theory may explain why kaempferol-3,4′-di-*O*-β-diglucoside showed no effect on hepatic xanthine oxidase activity.

Uricosuric drugs may go beyond their hypouricaemic effect. Endothelial dysfunction is closely linked to hyperuricaemia and the development of this condition predisposes to several other morbidities such as hypertension, systemic inflammation, atherosclerosis, cardiovascular diseases and diabetes [1]. A uricosuric effect may be beneficial in preventing the development of endothelial dysfunction. Kang et al. [28] demonstrated through in vitro models the role of uric acid in inducing a decrease in nitric oxide and stimulating the growth of vascular smooth muscle cells (VSMCs), which are crucial factors in the pathogenesis of endothelial dysfunction. In this study, pre-treatments with probenecid lessened the negative effects caused by uric acid. Probenecid has the ability to inhibit the entry of uric acid into VSMCs. If the EE of *E. sativa* acts by the same uricosuric mechanism, they can be potential candidates for preventing endothelial dysfunction. Kang et al. [28] also suggested that the harmful action of uric acid occurs through C-reactive protein, and that uric acid in concentrations of 6–12 mg/dL in the blood has the ability to regulate its expression. The findings of Mazzali et al. [29] stimulated the discussion about uricosuric treatments for hypertension, as it was observed that uric acid induces hypertension via activation of the renin-angiotensin system and inhibition of nitric oxide synthase 1 (ONS1) expression. Benefits in type II diabetes could also be explored, with uric acid being identified as having a bidirectional relationship with insulin resistance [30]. Anzai et al. [31] proposed a model for the renal reabsorption of uric acid, where URAT1 and GLUT9 act together. URAT1 mediates the uptake of urate from the urinary lumen into the renal tubule, which is then transported from the intracellular medium to the blood by GLUT9. Most uricosuric drugs show an inhibitory effect on URAT1. Probenecid and benzbromarone are potent URAT1 inhibitors, but moderately inhibit GLUT 9. Thiazide diuretics are able to increase uric acid reabsorption via URAT1, and while URAT1 is influenced by organic anions, GLUT9 has a high affinity for hexoses [31]. Kaempferol-3,4′- di-*O*-β-glucoside has two hexoses in its structure, which may be related to its uricosuric effect via GLUT9 inhibition.

A dose-dependent effect was observed in relation to the hypouricaemic and uricosuric effect when the doses were reduced from 40 mg/kg to 125 mg/kg in the EE. Although treatment with 40 mg/kg of EE did not promote an uricosuric effect, a less pronounced hypouricaemic effect was observed. When reducing the dose to 10 mg/kg of EE, there was no change in the effect, so the treatment with 10 mg/kg was considered equivalent to that of 40 mg/kg of EE. This indicates that there are other anti-hyperuricaemic mechanisms in addition to the uricosuric and uricostatic pathways by which *E. sativa* extracts promote the hypouricaemic effect. These findings create room for pursuing new alternatives for the treatment of hyperuricaemia that do not act by uricostatic and/or uricosuric routes. Other mechanisms include supplementation of uricase analogues, inhibition of previous pathways to the formation of xanthine and hypoxanthine and increased intestinal extra-renal excretion [32,33]. Uricases tested on humans are known to have immunogenotoxicity problems when administered intravenously. However, a recent study has shown that uricase administered orally to pigs is able to increase intestinal elimination of uric acid [34]. These findings draw attention to a new type of thinking in relation to the discovery of new anti-hyperuricaemic drugs. Products with active uricases, such as plants, could become anti-hyperuricaemic herbal medicines. As it turns out, researchers in Iraq have been able to isolate uricase from *E. sativa* seeds [35]. However, there are no reports of in vivo testing of uricases from plants, and further studies are needed.

Data from ethnopharmacological studies conducted with *E. sativa* in other countries report the use of the plant in folk and aboriginal medicine in the treatment of hypertension and diabetes [6,7]. In a complementary way, Hetta et al. [4] reinforced the proposition that *E. sativa* exhibits an antidiabetic action. Some works written in ancient Greece reported diuretic and renal benefits of the rocket plant. Such reports are confirmed by studies that indicated the nephroprotective activity of *E. sativa* against toxicity induced by pesticides [5]. From an ethnopharmacological point of view, several studies have shown similarities in the medicinal uses and biological activities of *E. sativa*, suggesting that, in general, the activities found for this plant are similar in different cultivars; however, this cannot be considered as a rule. Bell et al. [20] drew attention to the fact that *Eruca* species are still in an evolutionary process and, as a result, their phytochemical profiles may vary in composition and concentration. Interferents such as light, environmental stresses, temperature and genetic factors can also influence the chemical composition between varieties. The discovery of the uricosuric effect of kaempferol-3,4′-di-*O*-β-glucoside gives room for thinking towards the standardisation of the anti-hyperuricaemic activity of the EE of *E. sativa* leaves based on its flavonoid content. Therefore, doses of EE would be adjusted in accord with kaempferol-3,4′-di-*O*-β-glucoside concentration since it is a biological and a chemical marker.

## 3. Materials and Methods

### 3.1. Reagents, Chemicals and Analytical Instruments

Reagents: ethanol PA (Quemis^®^, Jundiaí, Brazil), hexane PA (Neon^®^, Suzano, Brazil), chloroform PA (Synth^®^, Diadema, Brazil), ethyl acetate PA (Qhemis^®^, Jundiaí, Brazil), methanol PA (Dynamic^®^, São Paulo, Brazil), acetonitrile (JTBaker^®^, Radnor, PA, USA), methanol (JTBaker^®^, Radnor, PA, USA), deuterated dimethylsulfoxide, diphenylboryloxyethylamine (Sigma-Aldrich, St. Louis, MI, USA), polyethylene glycol (Sigma-Aldrich), potassium oxonate (Sigma-Aldrich), uric acid (Sigma-Aldrich), Tween 80 U.S.P (Synth^®^, Diadema, Brazil), distilled water, saline solution (Sequiplex, Aparecida de Goiânia, Brazil), dimethyl sulfoxide (DMSO; Vetec, Duque de Caxias, Brazil), hydrochloric acid PA (Chomoline, Diadema, Brazil), monobasic potassium phosphate (Vetec), dibasic sodium phosphate (Vetec), dibasic sodium phosphate hepatic hydrate (Vetec), phosphoric acid 85% PA (Vetec), Brilliant Blue G-250 (Sigma-Aldrich), anaesthetics (injectable Dopalen; Ceva Santé Animale, Libourne, France), xylazine (injectable Dopaser; Hertape Calier, Juatuba, Brazil), benzbromarone (Sigma-Aldrich), probenecid (Sigma-Aldrich) and allopurinol (Sigma-Aldrich). For the determination of uric acid, the monoreagent uric acid kit was supplied by Bioclin (Belo Horizonte, Brazil). One-millilitre syringes (BD Plastipak, Jersey City, NJ, USA) and hypodermic needles (BD Precision Glide, Jersey City, NJ, USA) were used to administer solutions, treatments and anaesthesia to rats. Reverse-phase silica gel (KP-C18-HS Biotage^®^,Uppsala, Sweden), Phenomenex, Luna^®^ C18, Aschaffenburg, Deutschland (5 µm, 250 × 4.6 mm), Shimadzu Shim, Canby, OR, USA, pack XR-ODS III C18 (2.2 µm; 2 × 150 mm), Millex^®^ filtration device (0.45 µm) (Merk, Darmstadt, Germany), Millipore polytetrafluoroethylene (Merk, Darmstadt, Germany) membrane (PTFE, 0.45 µm), Milli-Q^®^ (Millipore^®^) filtration system (Merk, Darmstadt, Germany), plates and CCD 10 × 10 Biotage ^®^ KP-SIL were used (Biotage^®^, Uppsala, Sweden).

Instruments: Shimadzu^®^ and Quimis^®^ balances, M-560 Buchi^®^ melting-point meter, R-210 rotary evaporator (Buchi^®^, Switzerland), MARCONI^®^ (Piracicaba, SP, Brazil) greenhouse model MA035, MARCONI^®^ knife mill, ultraviolet (UV) light at 254/366 nm (Blak-Ray), Bruker Avance III 400 MHz spectrometer (Madison, WI, USA), Waters^®^ model 2695 high-performance liquid chromatograph, Waters 2996 diode array detector (DAD), UHPLC-ESI-QTOF system (Oswaldo Cruz Foundation, René Rachou Research Center), Cary^®^ 50 Bio Varian UV-vis spectrophotometer, Australia—quartz cubes (10 × 10 mm, 3500 μL; 10 × 10 mm, 600 μL) were used in the absorbance readings. Additionally used were Sigma Laboratory Centrifuges^®^ 3K30 centrifuge, Germany, and a Microcen^®^ 16 centrifuge from Herolab. The biological materials collected were stored in the Brastemp^®^ Flex freezer at –20 °C or in the freezer at –80 °C. Unique ultrasonic, Shimadzu and Quimis scales, Motion^®^ II vortex (Logen Scientific) and Digimed DM20 pH meter were used.

### 3.2. Plant Material

Seeds of *E. sativa* Miller of the variety Selecta (Broad Leaf) were obtained from the company Horticeres. The cultivation was carried out in a hydroponic system in semi-covered non-acclimatised greenhouses in the municipality of Mario Campos, MG, Brazil. Supplementation was made using fertilizers based on N, K, P and Fe. Collection of plant material was made on 18 June 2018. Leaves of the rocket were washed with water, dried in an oven with air circulation at 38 °C and pulverised in a knife mill.

### 3.3. Preparation of the Ethanolic Extract (EE) from E. sativa Leaves

Dried and pulverised leaves (370.0 g) were extracted by continuous percolation with ethanol until the plant material was depleted. The solvent was eliminated in a rotary evaporator and the material kept under vacuum to obtain 66.0 g of crude dry extract.

### 3.4. HPLC Analyses

#### 3.4.1. Qualitative HPLC-DAD Analysis

The method was developed in order to obtain a chromatographic profile that would detect the greatest possible number of peaks with better resolution of the chromatographic signal baseline; the purity of the peaks was also considered. The method used by Martínez-Sánchez et al. [13] was taken as a starting point for the development of the analytical condition for the fingerprint of the EE. Analyses were conducted using a Phenomenex column, Luna^®^ C18 (5 µm, 250 × 4.6 mm). The analysis temperature was set to 30 °C, flow rate at 1.0 mL/min and volume injection of 20 µL in all injections. Several conditions were tested to select the mobile phase (gradient elution with different proportions of water/methanol, water/acetonitrile, and acidified water/acidified acetonitrile). Thus, the mobile phase was composed of solvent A (ultrapure water/formic acid 0.1%) and solvent B (acetonitrile/formic acid 0.1%). The HPLC gradient was started with 100% of solvent A, reaching 80% A at 10.0 min, 50% A at 25.0 min and 0.0% A at 40.0 min, finally returning to the initial conditions at 45.0 min, which were maintained until 50 min. The detector was adjusted to read from 200–400 nm. Empower^®^ software was used for data processing and all chromatograms were extracted at 264 nm.

EE diluted in methanol was analysed by HPLC-DAD and the absorption spectra of its major peaks were extracted and analysed. In addition, a kaempferol standard was injected separately under the same conditions at a concentration of 0.5 mg/mL.

#### 3.4.2. UHPLC-ESI-QTOF Analysis

The method used was adapted from Martínez-Sánchez et al. [13]. EE (10 mg) was diluted in 500 µL of DMSO. Then, 100 µL of this solution was diluted in 300 µL of methanol. The sample injection volume was 5 µL. The analysis was conducted in an ultra-efficient liquid chromatograph using a column (2.2 µm; 2 × 150 mm; C18) coupled to an ESI-Q-q-TOF mass detector adjusted in positive ion mode. Ionisation was conducted via electrospray ionisation (ESI) and fragmentation was by collision-induced dissociation (CID). The detection range was set from 100 to 1500 m/z. The nebulisation was carried out at 3 Bar, the drying temperature was 200 °C and the capillary voltage was adjusted to 4500 V. The mobile phase was composed of solvent A (ultrapure water/formic acid 0.1%) and solvent B (acetonitrile/formic acid 0.1%). The chromatographic run started with 0% of solvent B, reaching 10% B in 20 min, 50% B in 25 min and finally reaching 100% B in 40 min. The proportion of 100% B was maintained for 5 min, returning to initial conditions in 1 min and maintained for another 9 min. The oven temperature was 40 °C during analysis and the flow of 400 µL/min was constant. The compounds were identified by comparing the results obtained with the available literature and mass spectra libraries.

### 3.5. Isolation of the Major Flavonoid in EE

Fractionation of EE was conducted using the method proposed by Nazif et al. [36] with adaptations. EE (55.0 g) was dissolved in distilled water at 60 °C. The solution was kept refrigerated overnight and then filtered on paper resulting in an insoluble residue (IE; 21.678 g) and an aqueous filtrate (Aq1). Aq1 was subjected to liquid–liquid partition with chloroform followed by ethyl acetate. Each solvent extraction was evaporated separately in a rotary evaporator in vacuo furnishing fractions EECl (0.349 g) and EEaE (0.273 g), respectively. The second aqueous fraction (Aq2) was filtered to yield an inorganic solid IS (7.78 g) and a filtrate (Aq3). Aq3 was submitted to open-column chromatography packed with reverse-phase (C18) silica gel; the elution was performed by using decreasing proportions of distilled water and methanol, starting with 100% water. Fractionation was monitored by silica gel TLC and revelation with NP-PEG [37]. Fraction (C1) eluted with water/methanol (75:25 *v/v*) was revealed to have an intense orange fluorescence spot when sprayed with NP-PEG. Subsequent chromatographic fractionations were performed employing the same method to yield a yellow powder (YP, 15 mg, flavonoid isolated from EE) with melting range at 217.3–218.9 °C. A DMSO-d6 solution of YP was analysed by NMR spectroscopy by ^1^H NMR, ^13^C NMR, COSY, HSQC and HMBC and the structure was confirmed to be kaempferol-3,4′-di-*O*-β-glucoside.

### 3.6. Validation of a Quantitative Method of the Major Flavonoid in EE by HPLC-DAD

Validation was carried out using parameters based on RDC 166/17 and international legislation [38,39]. Linearity, precision, intermediate precision and accuracy tests were conducted and are summarised in Table 3.

Firstly, selectivity was evaluated considering the degradation of products in alkali and extreme acid conditions (*n* = 3). EE methanolic solutions (4 mg/mL) were added to an equal volume of a 1.0 mol/L HCl solution and kept at room temperature. After 21 h, the solutions were neutralised with 0.5 mol/L NaOH to produce the final concentration of 1 mg/mL of extract. For the alkali condition, EE methanolic solutions were submitted to 1.0 mol/L NaOH solutions for 18 h and neutralised with 0.5 mol/L HCl solutions. The percentage peak degradation of kaempferol-3,4′-di-*O*-β-glucoside was 11.5 ± 0.019% in acid conditions, whereas in alkali conditions 46.6 ± 8.735% degradation was found. Peak purity analyses of the chromatographic signal were evaluated by Empower^®^ software. In both conditions, the kaempferol-3,4′-di-*O*-β-glucoside peak was pure in the range of 200–400 nm.

Due to a lack of the reference standard (kaempferol-3,4′-di-*O*-β-glucoside) to validate a quantitative method, a standard of similar UV absorption characteristics (rutin) was chosen. EE solutions in methanol (1 mg/mL) containing increasing concentrations of rutin (0.01, 0.02, 0.03, 0.04 and 0.05 mg/mL) were prepared in triplicate on two different days. Resolution of kaempferol-3,4′-di-*O-*β-glucoside and rutin peaks were adequate and symmetry of both peaks was less than 1.5. The peak assigned to rutin was pure in all runs. Using Prisma^®^ software, statistical treatments were applied to the obtained results in order to evaluate linearity. In both days, the average curve generated presented r > 0.99 (two-tailed test). On day 1, the curve obtained was y = 3.364e + 0.007x − 44,233 (R^2^ = 0.9973) and on day 2, the curve was y = 3.422e + 0.007x − 53,351 (R^2^ = 0.9946). The residue analysis followed a random distribution and confirmed that the variance of the experimental errors was homoscedastic. No outliers were evidenced. Experimental errors analysis made by Shapiro–Wilk test confirmed the normality of the experimental errors on the two days (Day: *p* < 1–0.88; Day 2: *p* < 0.94). Dependency error analysis (0.8182 < 1000) carried out on Prisma^®^ confirmed the independence of the results. Three concentrations in the curves of each day were selected (lowest, medium, and highest) to evaluate precision (repeatability and intermediary precision) and accuracy. Precision was expressed by relative standard deviation (RSD) of the response and the accuracy as the percentage deviation from the nominal concentration. In the first day of analysis, RSD of the responses were 1.0% (lowest concentration), 3.5% (medium concentration) and 1.7% (highest concentration). On the second day, RSD of responses were 5.4, 6.9 and 2.4%. The limit of acceptance for precision was considered to be 7.3%, according to the analytical quality manual recommended by the Ministério da Agricultura e Pecuária [40]. In all concentrations, deviations from the nominal concentration were <3%. The limits of detection (LD) and quantification (LQ) were obtained from the ratio of the standard deviation of the intercept with the *y*-axis (*n* = 6) with the slope of the average analytical curve obtained. This ratio was multiplied by 3 to obtain LD (0.0019 mg/mL) and by 10 to obtain LQ (0.0059 mg/mL). Ultimately, the validated method was selective, linear, precise and accurate. With a 95% confidence interval, it was seen that there were no statistical differences between the slope coefficients of the curves generated on the two days and their intersections with the *y*-axis. Thus, the mean analytical curve of days 1 and 2, y = 3.39333e + 0.007x − 48791.8, was assumed for all data.

### 3.7. Quantification of Kaempferol-3,4′-di-O-β-glucoside by HPLC-DAD

Three stock solutions of EE at 4 mg/mL in methanol were prepared. From these, solutions with a theoretical concentration of 2 mg/mL in methanol were obtained. Kaempferol-3,4′-di-*O*-β-glucoside present in the solutions was quantified using the HPLC-DAD method, developed and validated. The mean analytical curve obtained was used to determine the concentration in the sample solutions. Results were then expressed in g of rutin equivalent/kg EE.

### 3.8. Anti-Hyperuricaemic Assay

#### 3.8.1. Animals

In vivo experiments were conducted in adult and male Wistar rats weighing 180–280 g and provided by the Central Bioterium of UFOP. The experimental protocol was approved by the UFOP Animal Use Ethics Committee (CEUA-UFOP, protocol number 7504230419) and were in accord with the NIH guide for the care and use of laboratory animals published by the US National Institute of Health [41]. The 12 h/12 h light/dark cycle was maintained ad libitum.

#### 3.8.2. In Vivo Hyperuricaemic Model

To evaluate the anti-hyperuricaemic activity, the model described by Murugaiyah and Chan [42], Ferrari et al. [43] and Bernardes et al. [44] was used. Animals were initially deprived of water and food for 12 h. Hyperuricaemia was achieved by treating animals with a single dose of potassium oxonate (200 mg/kg, i.p.) and uric acid (1 g/kg, i.g.). Thirty minutes after hyperuricaemia induction, treatments [EE, doses of 10, 40 and 125 mg/kg diluted in vehicle DMSO/Tween 80/water (1:1:8)] and drugs of reference [allopurinol 10 mg/kg, benzbromarone 10 mg/kg and probenecid 50 mg/kg diluted in the vehicle ethanol/Tween 80/water (10:20:70)] were administered. Each group was formed by six animals. The normal control group (with normal uricemia) did not receive potassium oxonate and uric acid. Instead, it was given saline intraperitoneally and vehicle orally. The hyperuricaemic control (model control) received only the vehicle used to dilute treatments after hyperuricaemia induction. After administration of treatments, the animals were placed in individual metabolic cages and received 100 mL of water ad libitum. Urine was collected in graduated tubes and water consumption measured for 5 h. The collected urine was used for the quantification of uric acid. At the end of the experiment, the animals were anaesthetised with a combination of ketamine and xylazine (240 and 60 mg/kg, respectively), intraperitoneally. Blood samples were collected from the abdominal aorta and a thoracoabdominal laparotomy was carried out. The blood was kept at room temperature until coagulation and then centrifuged at 3000× *g* for 15 min. The resulting supernatant was again centrifuged at 3000× *g* for 15 min to obtain the serum. Serum and collected urine were stored at −20 °C for further analysis. The livers (3.0 g) were removed, weighed and stored at −80 °C for later preparation of the homogenates.

#### 3.8.3. Uric Acid Assay

Serum and urine samples had their uric acid measured by a colorimetric technique using a kit (Bioclin, Brazil), following the manufacturer’s instructions.

#### 3.8.4. Liver Homogenate Preparation

Livers were thawed and crushed in a 50 mM phosphate buffer solution (5 mL). During crushing, the samples were kept on ice. The crushed livers were centrifuged at 3000× *g* at 4 °C for 15 min. The upper lipid layer was discarded, and the intermediate supernatants collected and centrifuged at 10,000× *g* at 4 °C for 1 h. The intermediate supernatant was collected to obtain the homogenates used for the quantification of total proteins and for the evaluation of the residual activity of xanthine oxidase.

#### 3.8.5. Total Protein Assay

Measurement of total proteins was performed using the Bradford method [45]. An analytical curve using increasing masses of albumin was constructed; the equation of the line obtained was y = 0.0064x + 0.0352; R^2^ = 0.992. A 50 mM phosphate buffer solution was used to zero the equipment. The blank was prepared by adding 100 µL of phosphate buffer to 5 mL of Bradford’s reagent, followed by homogenisation in a vortex apparatus. The samples were prepared by adding 100 µL of homogenate to 5 mL of Bradford’s reagent and then homogenizing in a vortex apparatus. The absorbances were recorded at 595 nm on a spectrophotometer after 2 min.

#### 3.8.6. Hepatic Xanthine Oxidase Activity Assay

Synthesis of uric acid from xanthine was monitored spectrophotometrically according to the method described by Hall et al. [46]. To prevent the conversion of uric acid to allantoin, 100 µL of each homogenate was added to test tubes with 5.4 mL of 1 mM potassium oxonate solution. The solution was then pre-incubated at 37 °C for 15 min. Hence, 1.2 mL of the 250 mM xanthine solution was added and incubated for 30 min at 37 °C. At 0 and 30 min after adding the xanthine, 0.5 mL of HCl solution (0.6 mol/L) was added to stop the reaction. The solutions were centrifuged at 3000 g for 5 min and the supernatants collected to read the absorbance in a spectrophotometer at 295 nm. The blank was adjusted using the 1 mM potassium oxonate solution. The quantification of the uric acid formed was made by the difference between the absorbance values at times 0 and 30 min, which was compared with the analytical curve (y = 1.6629x − 0.0045; R^2^ = 0.998). With the total protein values, it was possible to express the residual activity of xanthine oxidase as nanomoles of uric acid formed per minute per milligram of protein.

#### 3.8.7. Statistical Analysis

Results processing of the animal model was made on GraphPad^®^ Prism software, version 6.01 (USA). Standard deviations among groups did not present a Gaussian distribution when applied the test of Shapiro–Wilk. The variance was performed using One-Way analysis of variance (ANOVA) and means were significantly different among the groups (*p* < 0.0001). Equal variances tests were performed using the Brown–Forsythe test and standard deviations among the groups were not significantly different [47]. Post-tests were performed using the Dunnett test and group pairs were compared using the Tukey test. *p*-values ≤ 0.05 were considered statistically significant. The results were expressed as means and standard deviation.

## 4. Conclusions

Analysis carried out on the ethanolic extract of *E. sativa* showed the presence of flavonoids and glucosinolates previously reported in other studies with *E. sativa*. Kaempferol-3,4′-di-*O*-β-glucoside was determined as the main flavonoid in the ethanolic extract, and it was considered a chemical and biological marker for uricosuric activity. Anti-hyperuricaemic activity of extracts were mainly but not only due to uricosuric action. Other mechanisms are still to be elucidated. Finally, the results obtained so far, associated with data from ethnopharmacological studies, drew attention to the potential of *E. sativa* to control hyperuricaemia and the comorbidities associated with this pathology.

## Figures and Tables

**Table 1:** Substances identified by UHPLC-ESI-QTOF in the ethanolic extract of *Eruca sativa* leaves.

Chemical Compound	RT (min)	Experimental Mass Data ESI^+^	Reference
Glucosativin glucosinolate	1.0	328.1384; 166.0861	[10]
Leucine	1.4	182.0808; 268.1037; 132.1019	Fiocruz
Glucoraphanin glucosinolate	1.8	438.0554; 196.0457; 358.0987	[11,12]
Tryptophan	4.2	188.0702, 146.0601; 205.0968	Fiocruz
Kaempferol-3,4′-di-*O*-β-glucoside	12.6	611.1598; 449.1070; 287.0544	[13]
Angustione	14.5	197.1168; 179.1062	Fiocruz
Kaempferol-3-*O-*β-glucoside	15.1	449.1068; 287.0544	[13]
Kaempferol-3-*O-*(2-sinapoyl-β-glucoside) -4′-*O*-glucoside	16.2	817.2178; 817,2166; 655,1641; 369,1166; 207,0646	[14]
Erucamide	46.4	338,3423	Fiocruz

**Table 2:** NMR data of kaempferol-3,4′-di-*O*-β-glucoside (400 MHz, DMSO-*d*6, 300 K).

Peak	*d* C-13	C-n	*d* H HSQC	*d* H HMBC	H-n (*J*/Hz)
1	177.49	C-4	---	---	---
2	164.81	C-7	---	6.21; 6.45	---
3	161.20	C-4′	---	5.03; 8.12	---
4	159.24	C-9	---	6.45	---
5	156.55	C-5	---	6.21	---
6	155.53	C-2	---	8.12	---
7	133.72	C-3	---	5.48	---
8	130.63	C-2′,6′	8.12	8.12	H-2′,6′
9	123.76	C-1′	---	7.16	---
10	115.83	C-3′,5′	7.16	7.16	H-3′,5′
11	103.96	C-10	---	6.21; 6.45	---
12	100.86	C-1A	5.48	nd	H-1A (7.50)
13	99.97	C-1B	5.03	nd	H-1B (7.25)
14	98.93	C-6	6.21	6.45	H-6 (1.73)
15	93.85	C-8	6.45	6.21	H-8 (1.73)
16	77.62	C-5B	3.09	nd	H-5B
17	77.10	C-5A	3.39	nd	H-5A
18	76.52	C-3A	3.29	nd	H-3A
19	76.44	C-3B	3.22	nd	H-3B
20	74.21	C-2A	3.17	nd	H-2A
21	73.23	C-2B	3.27	nd	H-2B
22	69.91	C-4B	3.08	nd	H-4B
23	69.58	C-4A	3.19	nd	H-4A
24	60.87	C-6B	3.57; 3.33	nd	H-6a,b-B
25	60.63	C-6A	3.69; 3.48	nd	H-6a,b-A

nd—not defined.

**Table 3:** Linearity, repeatability, intermediate precision and accuracy of the HPLC-DAD quantification method of kaempferol-3,4′-di-*O*-β-glucoside in the ethanolic extract of *E. sativa* on days 1 and 2.

Day	Linearity (*n* = 3)	Concentration Level	RSD % (*n* = 3)	Accuracy % (*n* = 3)
1	Y = 3.364e + 0.007X − 44233 R^2^ = 0.9973	Lowest	1.0	102.7–99.0
		Medium	3.5	100.6–94.5
		Highest	1.7	98.5–96.9
2	Y = 3.422e + 0.007X − 53351 R^2^ = 0.9946	Lowest	5.4	101.0–92.7
		Medium	6.9	104.0–93.5
		Highest	2.4	99.4–96.9

## Data Availability

Not applicable.

## Sample Availability

Samples of the compounds are not available from the authors.
